# Chitosan, a Biopolymer With Triple Action on Postharvest Decay of Fruit and Vegetables: Eliciting, Antimicrobial and Film-Forming Properties

**DOI:** 10.3389/fmicb.2018.02745

**Published:** 2018-12-04

**Authors:** Gianfranco Romanazzi, Erica Feliziani, Dharini Sivakumar

**Affiliations:** ^1^Department of Agricultural, Food and Environmental Sciences, Marche Polytechnic University, Ancona, Italy; ^2^Department of Crop Sciences, Postharvest Technology Group, Tshwane University of Technology, Pretoria, South Africa

**Keywords:** antimicrobial activity, biopolymer, coating, induced resistance, natural fungicide

## Abstract

Chitosan is a natural biopolymer from crab shells that is known for its biocompatibility, biodegradability, and bioactivity. In human medicine, chitosan is used as a stabilizer for active ingredients in tablets, and is popular in slimming diets. Due to its low toxicity, it was the first basic substance approved by the European Union for plant protection (Reg. EU 2014/563), for both organic agriculture and integrated pest management. When applied to plants, chitosan shows triple activity: (i) elicitation of host defenses; (ii) antimicrobial activity; and (iii) film formation on the treated surface. The eliciting activity of chitosan has been studied since the 1990’s, which started with monitoring of enzyme activities linked to defense mechanisms (e.g., chitinase, β-1,3 glucanase, phenylalanine ammonia-lyase) in different fruit (e.g., strawberry, other berries, citrus fruit, table grapes). This continued with investigations with qRT-PCR (Quantitative Real-Time Polymerase Chain Reaction), and more recently, with RNA-Seq. The antimicrobial activity of chitosan against a wide range of plant pathogens has been confirmed through many *in-vitro* and *in-vivo* studies. Once applied to a plant surface (e.g., dipping, spraying), chitosan forms an edible coating, the properties of which (e.g., thickness, viscosity, gas and water permeability) depend on the acid in which it is dissolved. Based on data in literature, we propose that overall, the eliciting represents 30 to 40% of the chitosan activity, its antimicrobial activity 35 to 45%, and its film-forming activity 20 to 30%, in terms of its effectiveness in the control of postharvest decay of fresh fruit. As well as being used alone, chitosan can be applied together with many other alternatives to synthetic fungicides, to boost its eliciting, antimicrobial and film-forming properties, with additive, and at times synergistic, interactions. Several commercial chitosan formulations are available as biopesticides, with their effectiveness due to the integrated combination of these three mechanisms of action of chitosan.

## Introduction

**FIGURE 1:**
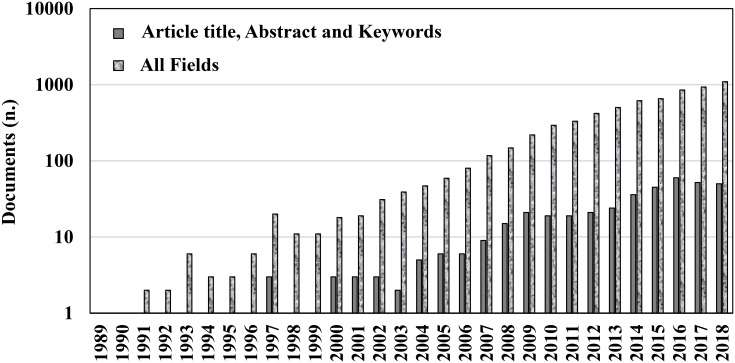
Number of documents available on Scopus through searches with keywords ‘chitosan’ and ‘postharvest’ in ‘Article title, Abstract, and Keywords’ or in ‘All fields’ published over the last 30 years (accessed on 6 November 2018).

Chitosan is the linear polysaccharide of glucosamine and N-acetylglucosamine units joined by β-1,4-glycosidic links and it is obtained by deacetilation of chitin through exposure to NaOH solutions or to the enzyme chitinase. Chitosan and chitin are naturally occurring polymers. For their biocompatibility and biosafety, their applications are widespread in many industries, such as cosmetology, food, biotechnology, pharmacology, medicine, and agriculture (Ding et al., 2013; Lei et al., 2014). In particular, chitosan has increasing interest in plant protection as a natural fungicide and plant defense booster, and meets the interest of many researchers, that used it to prolong the storage of an array of fruit and vegetables worldwide. Chitosan was the first compound in the list of basic substances approved in the European Union for plant protection purposes (Reg. EU 66 2014/563), for both organic agriculture and integrated pest management. A comprehensive review on the available data on the effectiveness of chitosan was published recently, for its preservation of fruit and vegetables, both alone and in combination with other treatments, and its mechanisms of action (Romanazzi et al., 2017). However, the increasing knowledge of this biopolymer (Figure 1) and the fast advances in basic and applied research in this field require a more focused and schematic update based on the last 5 years of investigations (2013–2018). The reader can then focus on specific aspects from the long list of other reviews that have appeared on the subject, among which some have focused on the applications of chitosan to fruit and vegetables (Bautista-Baňos et al., 2006; Bautista-Baòos et al., 2016; Zhang et al., 2011). When applied to plants, chitosan shows triple activity: (i) elicitation of host defenses; (ii) antimicrobial activity; and (iii) film formation on the treated surface. We will cover the recent information on these issues in the following sections, which is also listed comprehensively in the Tables, with examples of these applications.

## Effectiveness of Chitosan in the Control of Postharvest Decay of Fruit

The potential effectiveness of chitosan as a coating for fresh fruit was first proposed by Muzzarelli (1986). The first *in-vivo* application of chitosan on fruit was in the Josep Arul Laboratory, by Ahmed El Ghaouth, who produced a list of papers through the last decade of the last century. These included El Ghaouth et al. (1992), where they applied chitosan to strawberries and other fruit, both alone and in combinations with other potential biocontrol agents, which then contributed to the develop of some commercial formulations. Following these promising investigations, and with the growing need for alternatives to the use of synthetic fungicides, chitosan use became popular, and it was proposed to be part of a new class of plant protectants (Bautista-Baňos et al., 2006). Chitosan coatings have now been applied to numerous temperate and subtropical fruit, both alone and in combination with other treatments (Tables 1–3), with generally additive, and in some cases synergistic, effectiveness (Romanazzi et al., 2012).

**Table 1:** Postharvest chitosan treatments with other applications for storage decay of temperate fruit.

Fruit	Decay agent	Combination with chitosan	Reference
Table grapes	*Botrytis cinerea*	Salicylic acid	Shen and Yang, 2017
	General decay	Glucose complex	Gao et al., 2013
	*Aspergillus niger, Rhizopus stolonifer*	–	de Oliveira et al., 2014
	*Fusarium oxysporum*	–	Irkin and Guldas, 2014
	General decay	–	Feliziani et al., 2013a
	General decay	Ultraviolet-C	Freitas et al., 2015
	General decay	–	Al-Qurashi and Mohamed, 2015
	*Aspergillus niger, Botrytis cinerea, Penicillium expansum, Rhizopus stolonifer*	Menta essential oil	Guerra et al., 2016
	*Botrytis cinerea*	Salvia officinalis essential oil	Kanetis et al., 2017
Strawberry	*Botrytis cinerea*	Lavander and thyme essential oil	Sangsuwan et al., 2016
	General decay	Poeny extract	Pagliarulo et al., 2016
	*Penicillium expansum, Rhizopus stolonifer*	Olive oil processing waste	Khalifa et al., 2016
	Total microbial load	Natamycin, nisin, pomegranate, grape seed extract	Duran et al., 2016
	Total microbial load	Quinoa protein-chitosan and quinoa protein-chitosan-sunflower oil	Valenzuela et al., 2015
	Total microbial load	Sodium benzoate and potassium sorbate	Treviño-Garza et al., 2015
	*Botrytis cinerea*	Zataria multiflora essential oil	Mohammadi et al., 2015
	*Rhizopus stolonifer*	Cinnamon leaf essential oil containing oleic acid	Perdones et al., 2014
	General decay	–	Benhabiles et al., 2013
	General decay	Geraniol and thymol	Badawy et al., 2017
	General decay	Carboxymethyl cellulose, hydroxypropylmethyl cellulose	Gol et al., 2013
	*Botrytis cinerea*	Nanosized silver-chitosan composite	Moussa et al., 2013
	General decay	Beeswax	Velickova et al., 2013
	*Botryosphaeria* sp.	–	Wang et al., 2017
Pear	General decay	Cellulose nanocrystals	Deng et al., 2017
	General decay	Acylated soy protein isolate and stearic acid	Wu et al., 2017
Apple	General decay	Olive waste extracts	Khalifa et al., 2017, 2016
	*Penicillium expansum*	–	Darolt et al., 2016
	*Venturia inaequalis*	–	Felipini et al., 2016
	*Penicillium expansum*	–	Li et al., 2015
	Calyx senescence	V	Deng et al., 2016
Citrus	*Penicillium digitatum, Penicillium italicum*	Silver nanoparticles	Al-Sheikh and Yehia, 2016
	Colletotrichum gloeosporioides	*Pichia membranaefaciens*	Zhou et al., 2016
	*Penicillium digitatum, Penicillium italicum*	Cress and/or pomegranate extracts	Tayel et al., 2016
	*Penicillium digitatum*	Clove oil	Shao et al., 2015
	*Penicillium digitatum*	Cyclic lipopeptide antibiotics from *Bacillus subtilis*	Waewthongrak et al., 2015
	General decay	Carboxymethyl cellulose	Arnon et al., 2014
	Total microbial load	Silver and zinc oxide nanoparticles	Kaur et al., 2017
Peach	*Monilinia laxa*	Polyethylene terephthalate punnets containing thyme oil and sealed with chitosan/boehmite nanocomposite lidding films	Cindi et al., 2015
	General decay	γ-ray	Elbarbary and Mostafa, 2014
	*Monilinia fructicola*		Ma et al., 2013
	*Monilinia laxa, Botrytis cinerea, Rhizopus stolonifer*	–	Feliziani et al., 2013b
Sweet cherry	General decay	–	Pasquariello et al., 2015
	–	Hydroxypropyl methylcellulose	Shanmuga Priya et al., 2014
Plum	General decay	Ascorbic acid	Liu et al., 2014


**Table 2:** Postharvest chitosan treatments with other applications for storage decay of subtropical fruit.

Fruit	Decay agent	Combination with chitosan	Reference
Mango	Anthracnose (*Colletotrichum gloeosporioides*)	Spermidine	Jongsri et al., 2017
	Anthracnose (*Colletotrichum gloeosporioides*), stem-end rot (*L. theobromae* strains)	Lactoperoxidase system incorporated chitosan films	Kouakou et al., 2013
	Anthracnose	*Mentha piperita* L. essential oil	de Oliveira et al., 2017
	Anthracnose (*Colletotrichum gloeosporioides*), stem-end rot (*L. theobromae* strains)	Lactoperoxidase system incorporated chitosan films	Kouakou et al., 2013
	Anthrcanose	*Mentha piperita* L. essential oil	de Oliveira et al., 2017
Citrus	Green mold (*Penicillium digitatum*)	*Bacillus subtilis* ABS-S14	Waewthongrak et al., 2015
	Anthracnose (*Colletotrichum gloeosporioides*)	*Pichia membranifaciens*	Zhou et al., 2016
Avocado	Anthracnose (*Colletotrichum gloeosporioides*)	Thyme oil	Bill et al., 2014
Tomato	*Alternaria alternata*	Methyl jasmonate	Chen et al., 2014
	*Aspergillus niger, Rhizopus stolonifer*	Essential oil from *Origanum vulgare* L	Barreto et al., 2016
Pomegranate	*Penicillium* spp., *Pilidiella granati*	Lemongrass film	Munhuweyi et al., 2017


**Table 3:** Preharvest chitosan treatments with other applications for storage decay of temperate fruit.

Fruit	Decay	Combination with chitosan	Reference
Citrus	*Penicillium digitatum*	*Rhodosporidium paludigenum*	Lu et al., 2014
Peach	General decay	Calcium chloride	Gayed et al., 2017
Jujube fruit	*Alternaria alternata*	–	
Table grapes	*Botrytis cinerea*	Salicylic acid	Shen and Yang, 2017
	*Botrytis cinerea*	–	Feliziani et al., 2013a
Strawberry	*Botrytis cinerea* and *Rhizopus stolonifer*	–	Romanazzi et al., 2013; Feliziani et al., 2015
	*Botrytis cinerea*	–	Lopes et al., 2014
	General decay	–	Saavedra et al., 2016
Sweet cherry	*Monilinia laxa, Botrytis cinerea*, and *Rhizopus stolonifer*	–	Feliziani et al., 2013a


## Chitosan Eliciting Activity

**FIGURE 2:**
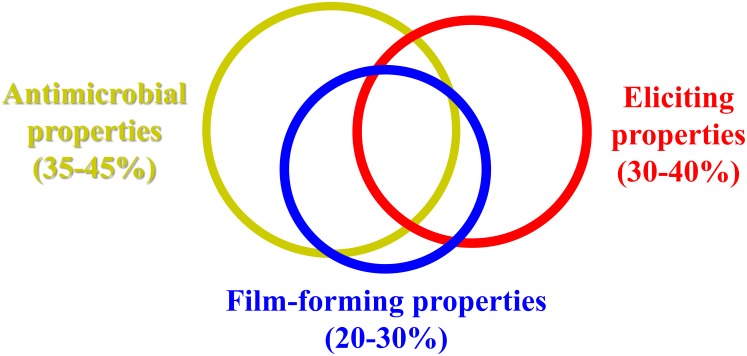
Proportion of antimicrobial, eliciting, and film-forming properties of chitosan.

Chitosan is known to elicit plant defences against several classes of pathogens, including fungi, viruses, bacteria and phytoplasma (El Hadrami et al., 2010). Moreover, in some studies, its eliciting activity was reported to be effective on pests (Badawy and Rabea, 2016). Based on our experience, the eliciting activity of chitosan accounts for 30 to 40% of its effectiveness in the control of postharvest decay of fresh fruit (Figure 2). The extent of this eliciting activity depends on the reactivity of the fruit tissues, and it is well known that fruit responses to stress decline with ripening (Romanazzi et al., 2016). This eliciting activity of chitosan has been studied since the 1990’s, which started with monitoring of the activities of enzymes linked to the defense mechanisms (e.g., chitinase) in different fruit (e.g., strawberries) (El Ghaouth et al., 1992). This was followed by investigations on other berries, citrus fruit and table grapes, among others. More recently, tools such as qRT-PCR and in recent years RNA-Seq (RNA-Sequencing) have allowed important information to be gained, first at the level of single gene expression, and then later at the level of global gene expression (Xoca-Orozco et al., 2017). This has provided good understanding of the multiple actions of chitosan applications and how they affect a number of physiological changes in fruit. As an example, the application of chitosan to strawberries at different times before harvest can affect the expression of a thousand or more genes (Landi et al., 2017). Some examples that have become available in the literature over the last 5 years are listed in Table 4, which deal with the physiological changes that can occur in chitosan-treated fruit, both when the biopolymer is applied alone, and when it is combined with other treatments. The eliciting activity of chitosan is particularly effective toward latent infections, as a more reactive fruit can stop the infection process, through a balance that resembles quorum sensing, which is well known for bacterial infections (Papenfort and Bassler, 2016).

**Table 4:** Physiological changes that can occur in fresh fruit after chitosan treatment, alone or in combination with other applications.

Fruit	Physiological change	Combination with chitosan	Reference
Apple	20 genes involved in defence responses, metabolism, signal transduction, transcription factors, protein biosynthesis, cytoskeleton.	–	Li et al., 2015
	Total phenolic, flavonoids, antioxidants, pigments, weight loss	Olive waste extract	Khalifa et al., 2017
Peach	Malondialdehyde content	γ-ray	Elbarbary and Mostafa, 2014
	Catalase, peroxidase, β-1,3-glucanase and chitinase	–	Ma et al., 2013
	Total soluble solids, weight loss, ascorbic acid content	Silver and zinc oxide nanoparticles	Kaur et al., 2017
	Color and fruit firmness	Polyethylene terephthalate punnets containing thyme oil and sealed with chitosan/boehmite nanocomposite lidding films	Cindi et al., 2015
	Fruit firmness, weight loss, total soluble solids, total phenolic content, and titratable acidity	Calcium chloride	Gayed et al., 2017
Plum	Fruit firmness, respiration rate, fruit color, polygalacturonase, superoxide dismutase, peroxidase, catalase, polyphenol oxidase, phenylalanine ammonia lyase and pectin methylesterase activities, superoxide free radicals, malondialdehyde content	Ascorbic acid	Liu et al., 2014
Sweet cherry	Malondialdehyde content and superoxide dismutase, catalase, ascorbate peroxidase, polyphenol oxidase, guaiacol peroxidase lipoxygenase activities	–	Pasquariello et al., 2015
Strawberry	Over 5000 differently expressed genes	–	Landi et al., 2017
	18 defence genes	–	Landi et al., 2014
	Fruit color	–	Feliziani et al., 2015
	Fruit firmness, anthocyanin and total phenol content	–	Saavedra et al., 2016
	Weight loss, titratable acidity, pH, total soluble solids, total phenols, anthocyanin and ascorbic acid content, activity of polygalacturonase, pectin methyl esterase, β-galactosidase and cellulose	Carboxymethyl cellulose, hydroxypropylmethyl cellulose	Gol et al., 2013
	Weight loss	Lavander and thyme essential oil	Sangsuwan et al., 2016
	Titratable acidity, soluble solids content	–	Benhabiles et al., 2013
	pH and soluble solids content	Natamycin, nisin, pomegranate, grape seed extract	Duran et al., 2016
	Weight loss, ascorbic acid	Poeny extract	Pagliarulo et al., 2016
	Weight loss, respiration rate, skin and flesh color, firmness, pH, titratable acidity, soluble solids content, reducing sugars content	Beeswax	Velickova et al., 2013
	Weight loss, firmness, color and total soluble solids content	Sodium benzoate, potassium sorbate	Treviño-Garza et al., 2015
	Weight losses, total soluble solids and titratable acidity	Olive waste extract	Khalifa et al., 2016
	Allergen-related gene	–	Petriccione et al., 2017
Table grapes	Phenylalanine ammonia lyase, chitinase, and β-1, 3-glucanase, phenolic compounds, respiration rate, weight loss, total soluble solids, titratable acidity	Salicylic acid	Shen and Yang, 2017
	Total phenols, flavonoids and ascorbic acid content, activities of peroxidase, polyphenoloxidase, polygalacturonase, and xylanase, fruit firmness	–	Al-Qurashi and Mohamed, 2015
	Fruit color	–	Irkin and Guldas, 2014
	Weight loss, titratable acidity, pH and soluble solids content, resveratrol content	Ultraviolet-C	Freitas et al., 2015
	Weight loss, soluble solids content and titratable acidity	Salvia officinalis essential oil	Kanetis et al., 2017
	Firmness, titratable acidity, soluble solids, color, weight loss	Menta essential oil	Guerra et al., 2016
	Total soluble solids, ascorbic acid content, titratable acidity, weight loss, respiration rate, activities of peroxidase and superoxide dismutase	Glucose complex	Gao et al., 2013
	Titratable acidity, soluble solids, color, firmness		de Oliveira et al., 2014
	Chitinase activity, quercetin, myricetin, and resveratrol content	–	Feliziani et al., 2013b
Citrus	Chitinase and phenylalanine ammonia lyase	–	Lu et al., 2014
	640 differentially expressed genes, many involved in secondary metabolism and hormone metabolism pathways	–	Coqueiro et al., 2015
	Fruit firmness, weight loss, total soluble solids	Carboxymethyl cellulose	Arnon et al., 2014
	Peroxidase and phenylalanine ammonia-lyase	Cyclic lipopeptide antibiotics from *Bacillus subtilis*	Waewthongrak et al., 2015
	Contents of chlorophylls and total carotenoids		
	Phenylalanine ammonia-lyase, β-1,3-glucanase, chitinase		
Jujube	Fruit firmness, cellulase, pectinase	–	Guo et al., 2017
Pear	Total phenolic and flavonoid contents, superoxide dismutase, peroxidase and catalase activities, total antioxidant activity	Calcium chloride	Kou et al., 2014a
	Malic acid-metabolising enzymes and related genes expression	Calcium chloride	Kou et al., 2014b
Mango	Peroxidase (POD) and polyphenol oxidase (PPO) gene expression	–	Gutierrez-Martinez et al., 2017
Kiwifruit	Induced gene expression and increased enzymatic activity of catalase, superoxide dismutase and ascorbate peroxidase	–	Zheng et al., 2017


## Chitosan Antimicrobial Activity

Numerous studies on chitosan inhibitory activities toward numerous microrganisms have been carried out since the first report of almost half a century ago (Allan and Hadwiger, 1979). The antimicrobial activities of chitosan against a wide range of plant pathogens have been confirmed by any of *in-vitro* and *in-vivo* studies. The antimicrobial activity of chitosan is one of its main properties, and this depends on the concentration at which it is applied. In the control of postharvest decay of fresh fruit, the antimicrobial activity can account for 35–45% of its effectiveness, as an antifungal barrier on a fruit inhibits the germination of fungal spores and slows down the rate of decay-causing fungi of already infected fruit, both latently and actively (Figure 2). A standard application rate of chitosan to provide a significant control of postharvest decay of fruit and vegetables can be considered 1%, except for the control of *Penicillia*, where higher concentrations may be needed to provide a good effectiveness. The degree of deacetylation and the molecular weight of chitosan characterize its properties, such as the number of positively charges of amino groups and therefore, its electrostatic interactions with different substrate and organisms at different pH. Chitosan with a higher degree of deacetylation, which has greater numbers of positive charges, would also be expected to have stronger antibacterial activities. On the other hand, numerous studies have generated different results relating to correlations between the chitosan bactericidal activities and its molecular weight (Romanazzi et al., 2017). In addition, there are many differences between the chitosan antifungal and antibacterial activities and several mechanisms relating to these remain still unclear and further researches are needed (Romanazzi et al., 2017).

## Chitosan Film-Forming Properties

Once applied to a plant surface by dipping or spraying, chitosan can form an edible coating, the properties of which (e.g., thickness, viscosity, gas, and water permeability) greatly depend on the acid in which the biopolymer is dissolved. The film-forming properties of chitosan account for 20–30% of the chitosan effectiveness in the control of postharvest decay of fruit and vegetables (Figure 2). Coating produces a barrier for gas exchanges and reduced respiration, and slows down fruit ripening. Of note, a less ripe fruit is less sensitive to postharvest decay.

## Toward Large-Scale Commercial Applications

When first used in experimental trials, chitosan needed to be dissolved in an acid (e.g., hydrochloric acid, acetic acid, which were among the most effective ones; see Romanazzi et al., 2009), and then taken to the optimal pH (∼5.6) This approach can even take 1–2 days, and it is impractical for use by growers. More recently, several commercial chitosan formulations that can be dissolved in water have become available on the market to be used as a biopesticides (Table 5). Some of these are formulated as powders, and then the cost of shipping is lower (although still higher compared to most of the commercially available synthetic fungicides), although the chitosan needs to be dissolved in water, in some cases a few hours before its application. This makes chitosan more difficult to use, as the grower wants to use an alternative to synthetic fungicides in the same way as a commercial compound, such that it should have the same effectiveness. This objective can be achieved with liquid formulations, which have concentrations of 2–15%. In this case, the cost of shipping is higher, as the volumes are larger due to the amounts of water that travel with the chitosan. In tests of three different commercial products, even when used at the same concentration, differential effectiveness was seen (Feliziani et al., 2013a). The higher cost of chitosan treatment compared to standard applications might also induce companies toward the use of low doses (e.g., even well below 0.1%), Based on data in literature, the optimal dose is around 1%, while decreasing the concentration, the effectiveness declines. Furthermore, when the concentration of chitosan is decreased, its effectiveness also declines. However, applications to the plant canopy also need to take in account possible phytotoxic effects, mainly if repeated applications occur. This has been shown for grapevines (Romanazzi et al., 2016a), such that for these purposes a good concentration might be 0.5%. However, under some particular conditions, even low concentrations of chitosan (e.g., 0.02%) in a commercial formulation can be beneficial, such as for the improved storage of litchi (Jiang et al., 2018).

**Table 5:** Some chitosan-based commercial products that are available for control of postharvest diseases of fruit and vegetables.

Product trade name	Company (Country)	Formulation	Active ingredient (%)
Chito plant	ChiPro GmbH (Bremen, Germany)	Powder	99.9
Chito plant	ChiPro GmbH (Bremen, Germany)	Liquid	2.5
OII-YS	Venture Innovations (Lafayette, LA, United States)	Liquid	5.8
KaitoSol	Advanced Green Nanotechnologies Sdn Bhd (Cambridge, United Kingdom)	Liquid	12.5
Armour-Zen	Botry-Zen Limited (Dunedin, New Zealand)	Liquid	14.4
Biorend	Bioagro S.A. (Chile)	Liquid	1.25
Kiforce	Alba Milagro (Milan, Italy)	Liquid	6
FreshSeal	BASF Corporation (Mount Olive, NJ, United States)	Liquid	2.5
ChitoClear	Primex ehf (Siglufjordur, Iceland)	Powder	100
Bioshield	Seafresh (Bangkok, Thailand)	Powder	100
Biochikol 020 PC	Gumitex (Lowics, Poland)	Liquid	2
Kadozan	Lytone Enterprise, Inc. (Shanghai Branch, China)	Liquid	2
Kendal cops	Valagro (Atessa, Italy)	Liquid	4
Chitosan 87%	Korea Chengcheng Chemical Company (China)	TC (Technical material)	87
Chitosan 2%	Korea Chengcheng Chemical Company (China)	SLX (Soluble concentrate)	2


## Concluding Remarks

The effectiveness of chitosan application arises from the integrated combination of its three mechanisms of action. There are increasing consumer requests for fruit and vegetables to be free from residues of synthetic pesticides, such that the rules defined by the public administration have become more limiting in terms of the active ingredients allowed and the maximum residue limits. Also, large stores compete with each other to further reduce these limits, compared to the legal thresholds (Romanazzi et al., 2016b). These trends make the concept of the application of alternatives to synthetic fungicides more popular, and among these the main one that is already used in human medicine is chitosan, which is particularly welcomed by public opinion. These aspects have promoted further studies based on the multiple actions of chitosan on fruit and vegetables. Therefore, further increases in our knowledge are expected following the widespread practical application of chitosan due to the regulation of its use in agriculture and the interest of companies to promote chitosan-based products, with potential benefits for the growers, the consumers and the environment.

## Author Contributions

GR proposed the review, collected data on chitosan popularity over time and on commercial products, coordinated the authors, and wrote the article. EF collected papers on effectiveness of chitosan on temperate fruit and on the mechanisms of action in the tables, and helped with the writing. DS collected papers on effectiveness of chitosan on tropical fruit and on the mechanisms of action in the tables, and helped with the writing.

## Conflict of Interest Statement

The authors declare that the research was conducted in the absence of any commercial or financial relationships that could be construed as a potential conflict of interest.
